# The correlation between economic development and suicide rate — based on the global WHO and WB database

**DOI:** 10.3389/fpubh.2025.1596682

**Published:** 2025-09-05

**Authors:** Juncheng Lyu, Li Ding, Zhiming Zhou, Qingxiang Zhang, Xinquan Li

**Affiliations:** ^1^School of Public Health, Shandong Second Medical University, Weifang, China; ^2^Qufu Normal University, Qufu, China; ^3^Shandong Xiangbai Hospital Co., Ltd. Weifang Hospital, Weifang, China; ^4^Department of Respiratory Critical Care Medicine, Weifang No.2 People’s Hospital, Weifang, Shandong, China

**Keywords:** economic development, global perspective, hierarchical analysis, psychological strains, suicide rates

## Abstract

**Background:**

Although some studies have reported correlations between economic factors and suicide rates, there is a lack of systematic analysis from a global data perspective.

**Methods:**

The most recent suicide rates (from 2017 to 2019) were obtained from the World Health Organization (WHO). The economic variables were obtained from the World Bank (WB) website. Software was used to match the suicide rates and the related variables from the database, country by country. Statistical methods were used to analyze the correlations between them.

**Results:**

Economic indicators, such as national income level, GDP per capita, GNI per capita, and the Gini index, are negatively correlated with suicide rates. However, this correlation was not consistent across all countries, and the direction of the association varied depending on the income levels of the countries. GDP per capita, GNI per capita, and the consumer price index were negatively correlated with suicide rates in the low-income group. A statistically significant correlation between the consumer price index and the Gini index was found in the lower-middle- and upper-middle-income groups, respectively. GDP per capita, GNI per capita, and the consumer price index showed positive correlations, but the Gini index had a negative correlation with suicide rate in the high-income group.

**Conclusion:**

The correlations between economic indicators and suicide rates vary by national income level, playing different roles at different stages of economic development. Differentiated and targeted monitoring of economic indicators has practical implications for suicide prevention.

## 1 Background

Suicide is a serious public health concern globally, and each suicide is a tragedy that affects families, communities, and nations. Although economic development has progressed rapidly, the suicide rate has decreased worldwide in the past three decades, which was still 11.5 per 100,000 people annually in recent years (1). More than 800,000 people die by suicide every year in the world, and every 40 s, a person dies by suicide somewhere in recent years (2).

The causes of suicidal behavior are complex, encompassing individual, demographic, economic, social, cultural (3), and mental health factors. Previous research has explored the association between economic factors and suicide rates with mixed findings. Certain studies focused on individual countries suggest a connection between unemployment and suicide (4, 5) and that rises in GDP may coincide with lower suicide rates (6). Some studies have reported the correlation between economic development and suicide rate (7–10). The previous literature showed that economic uncertainty is positively associated with suicide when controlling for unemployment [coeff: 8.026; 95% CI: 3.692–12.360] (7). Unemployment was significantly related to suicide and should be targeted as a risk factor for suicide prevention interventions in Kazakhstan (10). Meda et al. found that every 1% increase in global unemployment rates is associated with a 1% upsurge in male deaths by suicide (RR = 1.01, CI 95% 1.00–1.01) with respect to female deaths, while higher GDP per capita reduced suicide rates, particularly in low-income countries (*β* = −0.15, *p* < 0.01) (11). In addition, many researchers have studied the economic recession on the suicide rate (12–15). However, the conclusions of previous studies remain controversial. For instance, Meda et al. have shown that macroeconomic factors such as unemployment and GDP per capita are strongly associated with suicide rates across 175 countries over 27 years (11). Additionally, Blasco-Fontecilla et al. explored the correlation between GDP adjusted for purchasing power parity and suicide rates in 10 WHO regions, finding varying relationships based on the level of economic development (16). Income inequality, measured by the Gini index, has been positively associated with suicide rates in studies such as Lee et al. (17), which analyzed economic correlates of violent death rates (including suicide) in 40 countries for the years 1962–2008, revealing a significant relationship between the logged Gini coefficient and the combined violent death rates. The consumer price index was negatively associated with the number of suicides for female deaths. These findings underscore the relevance of our chosen indicators in understanding the complex relationship between economic development and suicide rates on a global scale (17).

The previous literature has explored the correlation between unemployment and suicide rate; for example, meta-analysis showed that unemployment was associated with a significantly higher relative risk (RR) of suicide before adjusting for prior mental health [RR 1.58, 95% CI (1.33–1.83)]. After controlling for mental health, the RR of suicide following unemployment was reduced by approximately 37% (RR 1.15, 95% CI 1.00–1.30) (16). Economic recession and unemployment are associated with an increased risk of suicidal behavior at the population and individual levels. Additionally, personal financial problems, such as debt and financial strain, are associated with an increased risk of suicidal behavior and ideation at the individual level (18).

As for the socioeconomic status indicators and suicide rate, some studies have attempted to probe the correlation between them. Nicolas Raschke found that low income, unemployment, and financial difficulties were identified as risk factors for all suicidal behaviors (19). Claveria O. suggests that increases in lagged economic uncertainty, as well as in unemployment and economic growth, may lead to an increased risk of suicide (20). A recent systematic review (21) found that the risk of suicide relating to socioeconomic factors varied greatly by local areas and between studies using various socioeconomic indicators; with higher deprivation, higher unemployment, lower income, and lower education levels were more likely to have higher suicide risk. Sotiris Vandoros and Ichiro Kawachi found that economic uncertainty is positively associated with suicide; an increase in the uncertainty index by 1% is associated with an additional 11–24.4 monthly suicide rates in the US (7). Claveria, O et al. found that decreases in economic uncertainty have a greater impact on suicide mortality than increases (22). The recent research shows that the suicide rate had a persistent effect, which varied over time depending on the transition variable within different threshold intervals (23). Blasco-Fontecilla reported that the correlation between purchasing power parity (PPP)-adjusted GDP per capita and suicide rates has positive, negative, and no correlation at different levels of economic development countries (24). Some studies have found that a deprived economic situation increases the risk of suicide (3, 25, 26). While some argue against this, other researchers suggest that economic development and modernization may contribute to a higher suicide risk (27, 28). Additionally, some studies have reported that economic development was not accompanied by a reduction in suicide rates (12, 29, 30). Moreover, previous studies have almost always conducted research using data from one region (12) or country (31). There is a lack of systematic analysis based on global data and perspective.

Therefore, this research aimed to assess the correlations between economic development and suicide rates using global WHO and WB databases to address these inconsistencies. By analyzing data from 183 countries across 2017–2019, covering low- to high-income nations, this study provides a comprehensive global perspective on how economic indicators correlate with suicide rates instead of focusing on single countries or regions, offering insights into tailored prevention strategies. By categorizing countries based on income levels, this research aims to explore how this correlation varies across different economic strata. The study was guided by the following research questions: (1) What is the overall correlation between economic indicators and suicide rates globally? (2) How does this correlation differ across low-income, middle-income, and high-income countries?

## 2 Data and methods

### 2.1 Data collection

The data used in this study cover the years 2017 to 2019. This period was chosen because it represents the most recent complete and available dataset from both the WHO and the World Bank at the time of data collection, ensuring the timeliness and relevance of the analysis. Additionally, using a 3-year span ensures the analysis reflects the latest trends in suicide rates and economic development. The study utilized age-standardized suicide rates (per 100,000 population) for 183 countries from the WHO (2017–2019) and economic data for 266 countries from the World Bank. The variables related to economic development, such as national income level, gross domestic product (GDP) per capita (standard conversion into current US dollars), gross national income (GNI) per capita, total unemployment rate (% of the total labor force), consumer price index (annual %), and Gini index, were selected based on professional knowledge. These indicators were selected because they reflect crucial aspects of economic development that could potentially influence suicide rates. National income levels provide a broad view of a country’s economic standing, while GDP and GNI per capita offer insights into individual economic welfare. The unemployment rate captures labor market dynamics, and the consumer price index reflects inflation and its impact on purchasing power. Finally, the Gini index allows us to assess income inequality, a factor often associated with social stress and mental health challenges, which in turn may affect suicide rates. The economic variables of 266 countries were obtained from the World Bank (WB) website. Data integration was performed using software to match datasets, country by country, with manual checks to ensure accuracy. It should be noted that suicide data in some countries may be underreported due to cultural or legal reasons, which could affect the accuracy of our findings. However, the official WHO data, which are standardized and validated to the extent possible, provide a reliable overview.

### 2.2 Variable definitions and data integration

In the current study, some indicators that reflect economic development have been extracted based on the previous literature, which has been mentioned earlier in the Background section, and our professional sociology knowledge. These indicators reflect crucial aspects of economic development that could potentially influence suicide rates.

National income levels provide a broader view of a country’s economic standing, while GDP and GNI per capita offer insights into individual economic welfare. The unemployment rate captures labor market dynamics, and the consumer price index reflects inflation and its impact on purchasing power. Finally, the Gini index allows us to assess income inequality, a factor often associated with social stress and mental health challenges, which, in turn, may affect suicide rates. The detailed introductions to these indicators are as follows:

The national income levels were categorized into four groups based on World Bank criteria, which is based on gross national income per capita (GNI per capita). Per capita GNI = gross national income (GNI)/population in the middle of the year (32). These national income levels were recorded as follows: 1 = low income, 2 = lower-middle income, 3 = upper-middle income, and 4 = high income.

GDP per capita is the gross domestic product divided by the mid-year population. It was calculated with the weighted average method and the standard converted by official exchange rates for current US dollars (33).

GNI per capita is the gross national income divided by the midyear population, which was converted to US dollars using the World Bank Atlas method (33). To smooth fluctuations in prices and exchange rates, the Atlas method of conversion is used by the World Bank. This applies a conversion factor that averages the exchange rate for a given year and the 2 preceding years, adjusted for differences in rates of inflation between the country and major economically developed countries (33).

The total unemployment rate (% of the total labor force) refers to the proportion of the labor force that is without work but available for and seeking employment. This rate was calculated and model-estimated by the International Labor Organization (33).

The consumer price index reflects inflation (annual %), which means the annual percentage change in the cost to the average consumer of acquiring a basket of goods and services that may be fixed or changed at specified intervals, always yearly (33). The Laspeyres formula, developed by German statistician Laspeyres in 1864, is generally used to calculate the consumer price index by the International Monetary Fund and International Financial Statistics.

The Gini index measures the extent to which the distribution of income or consumption among individuals or households within an economy deviates from a perfectly equal distribution. The Gini index measures the area between the Lorenz curve and a hypothetical line of absolute equality, expressed as a percentage of the maximum area under the line (33). Thus, the Gini index of 0 represents perfect equality, while the Gini index of 100 implies perfect inequality. The data have been adjusted for household size, providing a more consistent measure of per capita income or consumption.

Since the data were gathered from different websites with varying database structures and sequences, software and merging methods were used to integrate the databases by the same field (country code). After integration, SAS software and manual method were used to inspect and check to make sure the integration was correct.

### 2.3 Ethical approval

This study was approved by the ethics committee of the affiliated university. Since the data were collected from public databases, no ethical issues arose. The research group registered on the World Bank and the World Health Organization websites via email and submitted an application for downloading scientific research data. After the application was approved, the open public data on the websites were obtained.

### 2.4 Statistical analysis

The $\bar{x}\pm s$ and median (quartile range, Q) were used to describe quantitative data, while n (%) was used for qualitative variables. The Spearman correlation and hierarchical analysis methods were used to assess the correlations between suicide rates and related variables. The statistical significance level for all analyses was set at *α* = 0.05. SPSS 21.0 and Excel 2012 software were applied to analyze and integrate the data.

## 3 Results

### 3.1 Description of the sample

The results showed that the sample of countries included 13.0% low-income, 25.0% lower-middle-income, 25.0% upper-middle-income, and 37.0% high-income countries. A slight decrease in the global suicide rate (from 10.43 ± 9.37 to 10.08 ± 8.70 per 100,000 population) was observed between 2017 and 2019 across 183 countries in this study. The mean and median gross domestic product (GDP) per capita increased by USD$848.32 and USD$435.99, respectively, over 3 years. Over the 3 years, the mean of the gross national income (GNI) per capita increased by USD$1,554.39, and the median increased by USD$557.68. The global unemployment rate decreased, with the mean and median falling by 0.39 and 0.27%, respectively. Although the consumer price index fluctuated slightly, it remained relatively stable over 3 years. The Gini index also showed a downward trend, with the mean and median decreasing by 0.11 and 0.85, respectively, from 2017 to 2019 (Table 1).

**Table 1:** Description of suicide rates and economic variables from 2017 to 2019.

Variables	*N*	$\bar{x}\pm s$/M (Q)/n (%)		
		2017 years	2018 years	2019 years
Suicide rate (/100,000)	183	10.43 ± 9.37 8.65 (7.33)	10.24 ± 9.04 8.19 (7.33)	10.08 ± 8.70 8.28 (7.14)
GDP per capita (US dollars)	257	16376.25 ± 24288.62 6587.09(18275.18)	17333.17 ± 25857.58 6967.38(19186.69)	17224.57 ± 25656.24 7023.08(18429.78)
GNI per capita (US dollars)	245	13338.71 ± 18247.69 5762.32(14060.00)	14225.99 ± 19397.76 6090.00(14615.00)	14893.10 ± 20045.59 6320.00(15080.0)
Total unemployment rate (%)	235	7.48 ± 5.32 5.80 (5.43)	7.25 ± 5.24 5.62 (5.25)	7.09 ± 5.13 5.53 (4.88)
Consumer price index (%),	225	4.59 ± 13.06 2.38 (3.02)	3.97 ± 7.48 2.53 (2.34)	4.59 ± 18.53 2.22 (2.05)
Gini index	73	35.53 ± 6.99 35.35 (10.75)	35.76 ± 6.88 35.30 (10.18)	35.42 ± 7.56 34.50 (12.20)
National income level	216	Low income: 18(13.00%), Lower middle income: 54 (25.00%), Upper middle income: 54(25.00%), High income: 80 (37.00%)		

Table 1 still demonstrated that the variation indicators (SD and quartile range) of suicide rate and economic development variables are large, which indicates that there are significant differences worldwide in suicide rate and economic development variables.

### 3.2 The correlation between economic variables and suicide rate

Because there were different types of variables, the Spearman correlation method was used to examine the correlation between economic variables and suicide rate. When the significance level was set to two-tailed, there was a statistically significant correlation between suicide rates and the Gini index (*p* < 0.001). Although no statistically significant correlation was found between suicide rates and national income level, GDP per capita, or GNI per capita, the *p*-values were all less than 0.10. There was no statistically significant correlation between suicide rate and total unemployment rate or consumer price index (both *p* > 0.20). Since the direction of the correlation coefficient could be determined by professional knowledge, a one-tailed significance level was used. The results of the correlation analysis are shown in Table 2.

**Table 2:** Spearman coefficient (r_s_) between economic variables and suicide rate (/100,000).

Variables	2017 years	*p*	2018 years	*p*	2019 years	*p*
	r_s_		r_s_		r_s_	
National income level	−0.127	0.043	−0.141	0.029	−0.141	0.029
GDP per capita (US dollars)	−0.124	0.050	−0.125	0.048	−0.140	0.031
GNI per capita (US dollars)	−0.129	0.042	−0.134	0.037	−0.143	0.029
Total unemployment rate (%)	−0.028	0.355	−0.036	0.318	−0.039	0.301
Consumer price index (%),	0.001	0.494	0.007	0.462	0.095	0.112
Gini index	−0.335	0.002	−0.337	0.001	−0.382	0.002

The results indicated a negative correlation (r_s_ ranging from −0.124 to −0.143) between suicide rates and national income level, GDP per capita, and GNI per capita (*p* ≤ 0.05). Moreover, a significant negative correlation was observed between suicide rate and Gini index (r_s_ ranging from −0.335 to −0.382, *p* ≤ 0.001). No statistically significant correlation was found between suicide rate and total unemployment rate or consumer price index (*p* > 0.05).

### 3.3 Correlations between economic variables and suicide rates in different national income groups

To reduce confounders and control for bias related to different income levels of countries, integrated 3-year data were used, and hierarchical analysis was conducted to analyze the correlation between economic variables and suicide rates across countries categorized by different national income levels. The results provided interesting findings. For the overall sample of countries, the suicide rate had a statistically significant negative correlation with GDP per capita, GNI per capita, and Gini index (*p* ≤ 0.05). In low-income countries, the suicide rate had significant negative correlations with GDP per capita, GNI per capita, and the consumer price index (*p* ≤ 0.05). For the lower middle-income group, only the consumer price index variable became statistically significantly correlated with suicide rate (*p* ≤ 0.05). In terms of the upper middle-income group, only the Gini index had a significant correlation with suicide rate (*p* ≤ 0.05). However, GDP per capita, GNI per capita, and consumer price index had statistically significant positive correlations, but the Gini index had a statistically significant negative correlation with suicide rate in high-income countries (*p* ≤ 0.05).

From a longitudinal perspective, the correlations between GDP per capita, GNI per capita, unemployment rate, consumer price index, and suicide rate shifted from negative to positive across countries ranging from low- to high-income levels. As national income increased, the correlation between the Gini index and suicide rates shifted from positive to negative. The results are shown in Table 3.

**Table 3:** Correlation of the economic variables on suicide rate in different national income groups.

National income group	GDP per capita		GNI per capita		Total unemployment rate		Consumer price index		Gini index	
	r_s_	*p*	r_s_	*p*	r_s_	*p*	r_s_	*p*	r_s_	*p*
Whole sample	−0.129	0.001	−0.135	0.001	−0.032	0.229	0.035	0.220	−0.352	<0.001
Low income	−0.220	0.030	−0.295	0.005	−0.164	0.068	−0.359	0.002	0.191	0.287
Lower middle income	−0.088	0.136	−0.083	0.148	0.045	0.292	−0.225	0.003	−0.236	0.066
Upper middle income	0.099	0.119	0.060	0.238	0.064	0.226	0.146	0.051	−0.365	0.001
High income	0.150	0.030	0.156	0.025	0.008	0.460	0.340	<0.001	−0.178	0.041

## 4 Discussion

### 4.1 Correlation between economic indicators and suicide rates: overall trends

The current study showed that the suicide rate decreased by 0.35 for 100,000 persons in 3 years. Additionally, the main economic variables such as GDP per capita and GNI per capita increased slightly, but the total unemployment rate and Gini index showed a mild downward trend, and the trend of the consumer price index remained relatively stable.

The correlation analysis indicated that national income level, GDP per capita, GNI per capita, and the Gini index were negatively correlated with suicide rates annually. The negative correlation between national income level, GDP per capita, GNI per capita, and suicide rate can easily be explained. Generally, better economic development is associated with higher national income levels, GDP per capita, and GNI per capita (34, 35). Economic development leads to improved living conditions, better material security, and enhanced social welfare. Economic development usually makes it easier to achieve life goals and ideals, which can lead to reduced psychological stress and lower suicide rates, as confirmed by numerous previous studies (36, 37). However, the current study showed that the Gini coefficient was negatively correlated with suicide, which is noteworthy and warrants further investigation. The Gini index measures the extent to which the distribution of income or consumption among individuals or households within an economy deviates from a perfectly equal distribution (33). The Gini index provides a convenient measure of the degree of inequality. Theoretically speaking, a higher Gini index indicates high income inequality, which would lead to an increase in the suicide rate. This study, however, found a negative correlation, which is different from previous research (29). From a social perspective, higher income inequality generally fosters relative deprivation and social fragmentation, which may increase psychological distress. However, in the countries examined in this study, it seems that such effects may be offset by robust social safety nets and strong community support systems. Shah and Chatterjee reported a significant negative association between the Gini coefficient and older adults suicide rates across nations, indicating that, in some contexts, higher inequality might coexist with protective mechanisms that reduce suicide risk (38). Economically, the negative correlation could reflect scenarios where economic growth, even if it widens income gaps, leads to improvements in overall living standards, including enhanced access to healthcare, education, and other services. Such improvements could collectively reduce the suicide rate by alleviating some of the pressures associated with inequality. They may be related to other important confounding factors, which need to be further explored.

This study found a negative correlation between unemployment and suicide rate, but this correlation was not statistically significant, unlike previous studies (15, 39). This result verified that unemployment and suicide rates have a complex, not a stable, association modulated by the economic cycle and other factors (12, 40).

### 4.2 Differential impacts of economic indicators on suicide rates across income levels

Analysis of the combined 3-year dataset still reflected negative correlations between GDP per capita, GNI per capita, the Gini index, and suicide rates, which is consistent with the yearly analyses.

However, the results from the hierarchical analysis of different income groups were varied and intriguing, potentially offering new insights for further analysis and interpretation.

#### 4.2.1 Lower-middle and upper-middle-income countries

For lower-middle-income and upper-middle-income countries, GDP per capita and GNI per capita showed no statistically significant correlation with suicide rate. However, the consumer price index and Gini index variables became statistically significant in lower-middle-income and upper middle-income countries, respectively. Considering the reason, it may be that people’s income guarantees basic needs in these countries, rendering economic income less decisive in their lives. In low-income countries, an obvious change in the consumer price index or inflation easily leads to mental and behavioral changes in people. The reason may be the limited controllability of the unstable economic system and market, which have been elaborated above. In upper-middle-income countries, with the development of the economy, inequalities of income and wealth disparities become generally prevalent in those countries. Under these conditions, most residents would feel a strong sense of relative deprivation. High relative deprivation could make strong, unbalanced moods and psychological strains result in suicidal behavior (36, 37). The Gini coefficient reflects the degree of income equality. Generally, a higher Gini coefficient represents a more unequal income distribution, which may lead to suicide (29). Considering the current study, the reason may be the improvement of equity in other main aspects, such as education, mental disorder service, medical service, and social environment, decreasing the suicide rate, except for the equity of economic income. The Gini index, which is the equity of economic income, affects the suicide rate but yields to other major influencing factors in upper middle-income countries.

#### 4.2.2 Low-income countries

In low-income countries, GNI per capita and suicide rates are negatively correlated, while suicide rates and CPI are negatively correlated. It is well-known that, in low-income countries, people’s basic material needs are often not met, making economic and income levels critical factors in their lives. Higher GDP per capita and GNI per capita can greatly improve living conditions and quality of life. Despite their poverty and extremely poor living conditions, they usually experience high levels of mental wellbeing and low levels of psychological stress. The slight development of the economy could lead to obvious life improvement and then reduce the suicide rate. A consumer price index increase always leads to inflation or prices rising. Previous studies have shown a complex correlation between inflation and economic growth, with both positive and negative correlations reported (41). Generally speaking, the economic system is always immature in low-income countries, and the ability to control risks for economic development is not strong, which will inevitably bring about inflation and price rises. In theory, an increase in consumer price index should enhance the suicide rate. However, this study concluded that inflation reduced the suicide rate. In my viewpoint, inflation may be an intermediary phenomenon along with economic development. The negative correlation may be an illusion caused by the mediating effect, and other reasons need to be further explored.

#### 4.2.3 High-income countries

When it comes to high-income countries, GDP per capita, GNI per capita, and consumer price index showed a statistically significant positive correlation with suicide rate, while the Gini index had a statistically significant negative correlation with suicide rate. These findings indicate that, in economically developed nations, higher levels of these economic indicators are associated with higher suicide rates, which contrasts with the trends observed in lower-income countries. The underlying reasons for these associations are complex and may involve various socioeconomic factors, which need further investigation. In our opinion, the social expectations and demands of life increase and become higher on the spiritual level in high-income countries. The rapid economic development would lead to high social pressure (such as work pressure and pressure of competition) and more mental health problems. The gap between expectation and reality is widening, which is more likely to lead to an increase in suicide rates (36, 37). Previous literature still reported that higher levels of GDP (28) and social pressure caused by modernization might be associated with higher suicide risk in developed regions (27).

Comparing the relation coefficient of the Gini index with suicide among countries with different income levels, the current study found that, although there was no significant correlation in low-income countries, the Gini index was positively associated with suicide. In lower-middle, high-middle, and high-income countries, the Gini index was negatively correlated with the suicide rate. The different direction correlation may be caused by the wide difference in sample size among different income groups. It also further proves that, in countries whose economic development is low, the increase in economic income and equity of income would reduce the suicide rate. Economic level and income play a decisive role in low-income countries. However, in lower-middle, upper-middle, and high-income countries, economic income may no longer be the main influencing factor for suicide; there may be other more dominant influencing factors for suicide. Understanding the complex association and other contributing factors, such as political, social, and cultural influences (3), is also an important task in preventing suicide (15).

### 4.3 Limitation and implications for future research

The study does have several limitations that warrant consideration. First, the use of data from 2017 to 2019 indicates that our analysis predates the COVID-19 pandemic. The pandemic has undeniably reshaped global economic landscapes and had a profound impact on mental wellbeing, potentially limiting the direct applicability of our findings to the present day. Second, missing data, substantial variation in indicator quality across different official databases, and potential ecological fallacy may have introduced some bias into our results and complicated cross-country comparisons. Third, the correlation analysis allows us to identify associations, but it cannot establish causation, and other confounding factors (9) add complexity to determine the independent effects of economic indicators. To address these limitations, future studies may incorporate more recent data to capture emerging post-pandemic trends, and future research could benefit from the use of regression analysis to better control for potential confounding factors or longitudinal studies to examine the temporal dynamics of the associations we observed. Finally, the use of visual aids such as bar charts could enhance the clarity of our results presentation.

## 5 Conclusion

This study suggests that the correlation between economic indicators and suicide rates varies between specific indicators and national income levels. Globally, national income level, GDP per capita, and GNI per capita are generally negatively correlated with suicide rates, but in high-income countries, these indicators may show positive correlations, indicating that economic development has different implications for suicide prevention at various stages. The negative correlation with the Gini index may be influenced by economic development or confounding factors. The study highlights potential differences across income levels, suggesting that economic indicators may play distinct predictive roles in suicide prevention. The findings suggest that the correlation between economic development and suicide is complex and dynamic, varying by the stage of economic development. However, it is important to recognize that our research identifies correlations and cannot establish causation. The findings are preliminary and limited because of the lack of control for certain confounding variables.

Future research could delve into the mechanisms behind these varied correlations, such as the roles of social welfare systems, cultural factors, or mental health services, in mediating the impact of economic development on suicide rates. Whether high income levels in high-income countries lead to increased social pressure and expectations, thereby contributing to higher suicide rates, is a scientific hypothesis that warrants rigorous investigation. Differentiated monitoring of economic indicators might have practical implications for suicide prevention, which needs further exploration.

## Data Availability

The original contributions presented in the study are publicly available. This data can be found here: WHO, World Bank (https://www.who.int/publications/i/item/9789241564779).
